# Neurotropic RNA Virus Modulation of Immune Responses within the Central Nervous System

**DOI:** 10.3390/ijms23074018

**Published:** 2022-04-05

**Authors:** Christine Vazquez, Kellie A. Jurado

**Affiliations:** Department of Microbiology, University of Pennsylvania, Philadelphia, PA 19104, USA; christine.vazquez@pennmedicine.upenn.edu

**Keywords:** virus, CNS, interferon, immune signaling, evasion

## Abstract

The central nervous system (CNS) necessitates intricately coordinated immune responses to prevent neurological disease. However, the emergence of viruses capable of entering the CNS and infecting neurons threatens this delicate balance. Our CNS is protected from foreign invaders and excess solutes by a semipermeable barrier of endothelial cells called the blood–brain barrier. Thereby, viruses have implemented several strategies to bypass this protective layer and modulate immune responses within the CNS. In this review, we outline these immune regulatory mechanisms and provide perspectives on future questions in this rapidly expanding field.

## 1. Introduction

Immune responses within the central nervous system (CNS) are tightly controlled to avoid excessive immune activation and inflammatory states, while also protecting against invading pathogens, such as viruses. Microglia, astrocytes, and neurons communicate with one other via signaling processes to maintain CNS homeostasis and mediate appropriate immune responses. Several protective mechanisms are present to shield the CNS from viruses. Perhaps the most well-described blockade is the blood–brain barrier (BBB), a highly selective, vascularized semipermeable barrier composed of endothelial cells. The BBB is highly efficient in preventing foreign molecules and peripheral immune cells from reaching the brain. However, some viruses have evolved the neuroinvasive capacity to circumvent this level of protection and infect CNS tissue. Several reviews have highlighted immune responses in the CNS during RNA virus infection and are beyond the scope of this review (reviewed in [1,2,3]). Here, we aim to outline the strategies utilized by neurotropic RNA viruses to modulate immune responses specifically within the CNS, which may lead to neuropathogenesis.

## 2. Several RNA Viruses Cause Neurotropic Disease

**Figure 1:**
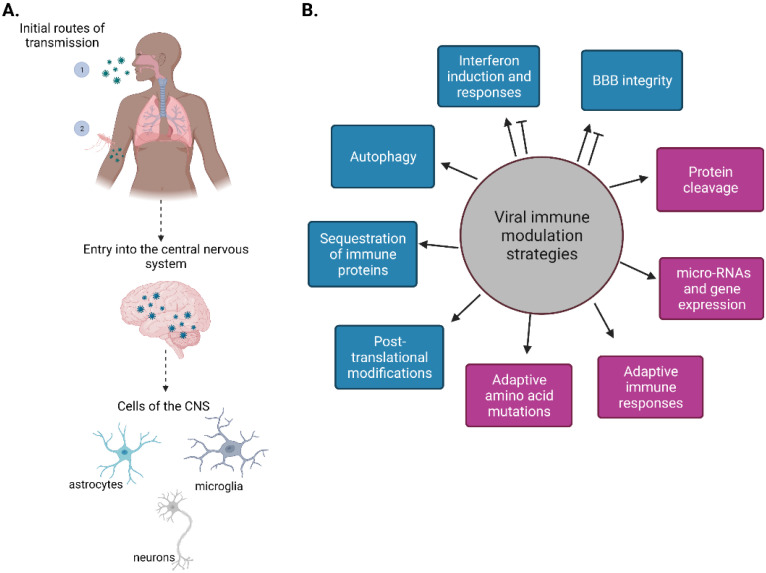
RNA viruses can be neurotropic. (**A**). Schematic of RNA virus entry into the central nervous system (CNS). Neurotropic viruses can infect human hosts via at least two routes of transmission: (1) respiratory droplets or (2) through the bite of an infected insect vector. Upon entry through the blood–brain barrier (BBB) and into the CNS, viruses can infect several CNS cell types, including astrocytes, microglia, and neurons. (**B**). Schematic of known immune modulation strategies enacted by viruses within the CNS. Arrows indicate activation while blunt-end arrows represent inhibition. Blue blocks indicate a strategy that can either increase or decrease interferon, while purple blocks indicate a strategy that decreases interferon. Created with BioRender.com.

Neurotropic viruses have the capacity to enter the nervous system and establish infection in the CNS, including in neurons (Figure 1A). Neurotropic viruses include members of the families *Picornaviridae* (poliovirus, non-polio enteroviruses, and coxsackie viruses), *Flaviviridae* (Japanese encephalitis virus (JEV), West Nile virus (WNV), dengue virus (DENV), and Zika virus (ZIKV)), *Rhabdoviridae* (Rabies virus (RABV)), *Togaviridae* (Venezuelan equine encephalitis virus (VEEV), Eastern equine encephalitis virus (EEEV) and Western equine encephalitis virus (WEEV)), *Paramyxoviridae* (mumps virus, measles virus (MV) and Nipah virus (NiV)), *Bunyaviridiae* (La Crosse virus and Rift Valley fever virus), and *Coronaviridae*(severe acute respiratory syndrome coronavirus 2 (SARS-CoV-2)). Although there are several DNA viruses and retroviruses that are neurotropic, such as herpes simplex virus, Epstein–Barr virus, or human immunodeficiency virus (highlighted in [4,5,6]), this review will focus on neurotropic RNA viruses causing diseases in humans. Sindbis virus and vesicular stomatitis virus are neurotropic in mice and therefore will not be discussed here. Table 1 highlights several neurotropic viruses and the CNS manifestations during viral infection. Below we outline the various immune antagonism strategies utilized by neurotropic RNA viruses (Figure 1B).

**Figure 2:**
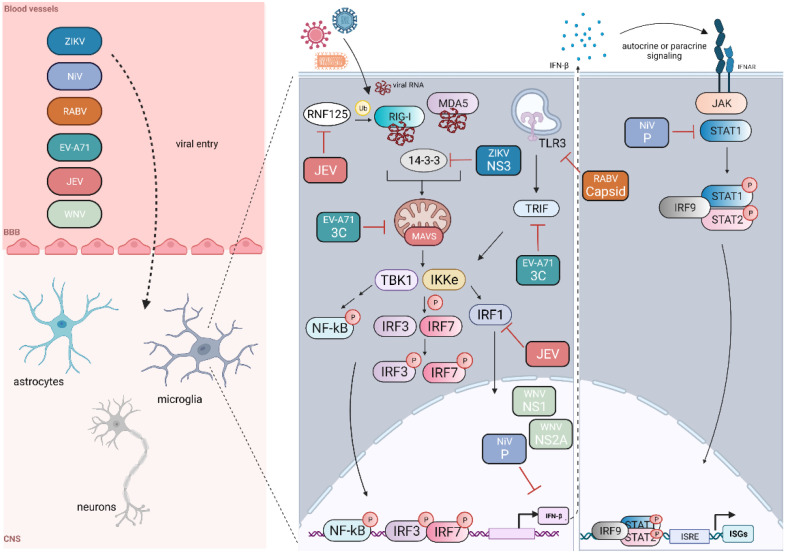
Neurotropic RNA virus evasion of IFN-β induction and responses in the CNS. Several RNA viruses can bypass the blood–brain barrier (BBB) through the circulatory system and enter the cells of the central nervous system (CNS). We also note that viral entry into the CNS may occur through other routes in addition to the circulatory vessels depicted within microglia; these viruses can block (red inhibitory arrows) components of the RIG-I signaling pathway, blocking IFN-β induction and subsequent JAK-STAT interferon signaling. Black arrows indicate subsequent steps within the signaling pathway or translocation from the cytoplasm to the nucleus. Created with BioRender.com.

Viruses may target and antagonize cellular processes and immune signaling pathways. These processes include regulation of subcellular localization, post-translational modifications, and autophagy. One of the most potent antiviral signaling molecules secreted by cells is the type I interferon (IFN), IFN-β. In a study examining the role of type I IFN signaling in organotypic brain cultures derived from SLAM-transgenic wild-type (WT) or IFN receptor (IFNAR)-depleted mice infected with MV, increased viral RNA copies were found in IFN-depleted conditions compared to the WT brain cultures [7]. This emphasizes the important role IFN plays in limiting viral burden with CNS tissue. Viral RNA receptors, kinases, and transcription factors, including IRF3 and NF-κB, stimulate the transcription of IFN-β. RNA viruses have implemented strategies to suppress interferon activation and downstream responses within cells of the CNS. Figure 2 depicts some interferon evasion mechanisms of a few of these viruses, although there are likely many more viruses and subversion strategies that have not been described or identified in the context of the CNS. We describe these mechanisms in detail below.

## 3. Immune Modulation Strategies

### 3.1. Regulation of Interferon Induction and Response

RNA virus infection is detected by pattern recognition receptors (PRR), which recognize essential viral features, such as the viral genome, and initiate downstream signaling cascades, ultimately leading to viral clearance through the function of antiviral immune molecules. PRRs include the retinoic-acid inducible gene I (RIG-I)-like receptors (RLR), Toll-like receptors (TLR), and nucleotide-binding and oligomerization domain (NOD)-like receptors (NLR) [8]. Cells of the CNS can preferentially utilize different PRRs to initiate signaling. Neurons signal through several antiviral PRR pathways, initiated by TLR3, RIG-I, and MDA5, leading to NF-κB- and IFN-β-specific immune responses. Viruses can circumvent this PRR-mediated signaling. In BE(2)-C/m cells, a differentiated human neuroblastoma cell line, WEEV blocks polyinosinic-polycytidylic (poly(I:C))-mediated activation of NF-κb and IFN-β promoters, but not signaling to the interferon-stimulated response element, suggesting that WEEV blocks interferon induction [9]. This inhibition is dictated by the viral capsid protein and occurs downstream of IRF3 activation. WNV is also able to block transfected poly (I:C)-stimulated ISRE and NF-κB promoter activity via the actions of its NS1 and NS2A viral proteins in BE(2)-C/m cells [9]. Additionally, in a ferret model of human NiV pathogenesis, NiV P, V, and W proteins can inhibit STAT1 responses to modulate the course of NiV disease in the brain, suggesting a role for immune responses in reducing neuropathogenesis [10]. Examination of the association between dysregulation of immune responses and neuropathogenesis is an important area of neurotropic RNA virus research. In a study examining whether type I IFN protects against WNV CNS infection, mouse primary WT superior cervical ganglion neurons were isolated and either pre-treated with IFN-α/β or mock-treated and subsequently infected with WNV [11]. IFN-α/β pre-treatment of neurons inhibited WNV-induced neuronal cell death [11]. Further, eight-to-ten-week-old WT- 129Sv/Ev mice intracranially infected with WNV showed no viral titer in the brain or spinal cord at 72 h post-infection compared to IFNAR-depleted mice [11], further supporting a role of IFN signaling in limiting viral disease burden.

Viral infection within the CNS can lead to the transcriptional induction of IFNs, antiviral cytokines, and inflammatory mediators, similarly to what is observed during RNA virus infection of other cell types. Neurons have long been considered poor IFN-producers, yet some studies have found that infected neurons can produce and respond to IFN-β [12,13]. Within neurons, viral infection also upregulates cytokine production [14]. Interestingly, different compartments within the brain have different immune signatures and susceptibilities to virus infection [15]. For instance, granule cell neurons are less susceptible to WNV, VEEV, and St. Louis encephalitis virus infection than cortical neurons as they express higher basal levels of several antiviral interferon-stimulated genes (ISGs), including *Ifi27* and *Rsad2* (*Viperin*) [14].

Viruses can also modulate negative mediators of IFN signaling. Previous studies have implicated a role for *ISG15* in negatively regulating IFN signaling by conjugating to RIG-I, resulting in RIG-I degradation [16,17]. In a genome-wide CRISPR-Cas9 knockout screen in human neural progenitor cells, Li et al. found that in these cells, when ISG15 protein expression is depleted, ZIKV replication is reduced, although the mechanism of this regulation is unknown [18].

### 3.2. Sequestration of Host Immune Proteins

Protein subcellular localization largely dictates protein function [19]. During RNA virus infection, several proteins relocalize to other compartments within the cell to (i) interact with other immune proteins, (ii) perform a separate function, or (iii) coordinate signaling pathways. For instance, the RNA sensor RIG-I is localized within the cytoplasm, where it can detect and bind to viral RNA. After RNA binding, RIG-I translocates to the mitochondria or the mitochondrial-associated ER membranes, where it then interacts with an innate immune adaptor protein, MAVS. Although many of the immune protein localization profiles have been explored in non-CNS cell types, several have also been observed in mouse CNS models or human neuron-like cell lines. In mouse brain sections and in the neuroblastoma cell line N2a, RIG-I is localized within the cytoplasm [20]. TLR3, another PRR, is localized within endosomal compartments and can respond to poly (I:C) [21]. As such, one strategy utilized by viruses is sequestering immune proteins away from their normal subcellular localization. Human neuronal cells express TLR3, and at steady state in uninfected neuronal cells, TLR3 is localized intracellularly within early and late endosomes [22]. RABV sequesters TLR3 by inducing the formation of cytosolic protein aggregates called Negri bodies. The viral nucleocapsid protein is found within these Negri bodies and is thought to aid in viral replication. During RABV infection, TLR3 maintains some endosomal localization; however, two days post-infection, TLR3 relocalizes to the center of these perinuclear Negri bodies. Accordingly, TLR3−/− mice have reduced susceptibility to RABV infection, suggesting that these TLR3-containing Negri bodies are necessary for virus infection [22]. However, a mechanistic understanding of TLR3 sequestration into these vesicles or the interactions occurring within these bodies is not characterized.

Subcellular localization and signaling processes rely on the function of molecular chaperones, proteins that assist or interact with other proteins in aspects of their function or conformation [23]. One important molecular chaperone in the RLR signaling pathway, mediated by the sensors RIG-I and MDA5, is the protein 14-3-3ɛ. The protein 14-3-3ɛ belongs to the 14-3-3 chaperone family, which has crucial roles in many cellular processes, including apoptosis and protein trafficking [24]. During RNA virus infection, 14-3-3ɛ binds to cytoplasmic RIG-I, moving it from the cytoplasm to signaling platforms on organelles, such as the mitochondria [25,26]. During ZIKV infection of SVGA cells, immortalized human fetal astrocyte cells, the ZIKV protein NS3 inhibits the translocation of RIG-I from the cytosol to the mitochondria. ZIKV NS3 binds to 14-3-3ɛ and competes with RIG-I for 14-3-3ɛ binding, blocking downstream interferon signaling. Another 14-3-3 subunit, 14-3-3η, which serves as a molecular chaperone for MDA5-mediated signaling, is also inhibited by NS3 [25].

Nipah virus infection relocalizes immune proteins as an immune evasion mechanism. In NiV-infected endothelial cells, the viral P and V proteins sequester STAT1 in the cytoplasm after binding. Yet, in NiV-infected human M17 neuroblastoma cells, the viral W protein sequesters STAT1 in the nucleus, suggesting that the virus can differentially modulate immune responses depending on cell type [27]. The NiV P, V, and W proteins contain an N-terminal STAT1 binding domain, and in vitro analysis has identified seven amino acid mutations within the P protein that abrogate STAT1 binding and IFN activation: Y116E, G121E, G127E, G135E, G125E, S130A, and S131A [4]. In a subsequent study, these mutations, combinations of mutations, or region deletions were introduced in a recombinant NiV construct followed by infection of 6–8-month-old ferrets. Examination of the ferret brains revealed that ferrets intranasally infected with the P_Δ116–135_ recombinant virus had severe neurological pathology, including meningitis and neuroinvasion with viral antigen present in the hippocampus, brainstem, cerebellum, and cerebrum [10]. These in vivo data demonstrate that the residues present within the 116–135 region of the P protein are important for limiting viral neuroinvasion and neuropathogenesis, most likely by interfering with IFN activation.

### 3.3. Post-Translational Modifications and Interactions with Modifying Enzymes

Post-translational modifications, such as phosphorylation, acetylation, and ubiquitination, play integral roles in maintaining protein stability and function. During antiviral signaling in non-CNS cell types, several key immune proteins undergo post-translational modifications. For instance, the transcription factors TBK1, IRF3, and STAT1 are phosphorylated for their activation and nuclear translocation. RIG-I is ubiquitinated via activating K63 and deactivating K48 ubiquitin linkages that modulate its conformation and interactions with other immune proteins, and ultimately its function. These modifications within the context of cell types of the CNS are less characterized but may likely occur in the same manner, as several CNS cells express the antiviral immune signaling proteins and pathways present in non-CNS cell types [5,12,28,29].

Microglial regulation in the CNS relies on several E3 ubiquitin ligases, which, in turn, maintain CNS homeostasis, limit inflammation and neurodegeneration, and orchestrate antiviral immune responses [30,31]. Peli1 is an E3 ubiquitin ligase expressed in microglia that negatively regulates type I IFNs by promoting the degradation of TRAF3 and the K48-linked ubiquitination of c-Rel [32,33,34]. However, Peli1 may also positively regulate NF-κB activation in neurons and microglia via interactions with the kinase RIPK1 [35]. WNV usurps Peli1 function by promoting its expression during infection, which aids in WNV entry and replication in peripheral myeloid cells and resident CNS cells [35,36]. Further, microglia have also been found to contribute to WNV-induced encephalitis by promoting the influx of inflammatory cytokines and chemokines. Microglia and immune signaling are activated during WNV, yet viral loads are not proportionately decreased, suggesting that either these immune responses are ineffective at limiting virus replication, WNV can evade uncharacterized innate signaling responses, or that WNV may regulate additional immune responses, such as T cell-mediated responses within the CNS to continue replicating. Future studies should parse apart the seemingly paradoxical roles of Peli1 and microglial activation during WNV or other neurotropic virus infections.

Viral proteins may become glycosylated or ubiquitinated. This adds yet another defense strategy for immune evasion in the viral arsenal. The amino acid sequence of the non-structural protein 1 (NS1) of flaviviruses is highly conserved amongst several family members, including DENV, WNV, and Yellow Fever virus (YFV) [37]. NS1 exists in multiple isoforms, localizes to several cellular compartments, and plays important roles in viral replication and immune system engagement [38]. Although what coordinates these functions of NS1 is largely unknown, it is known that NS1 is heavily glycosylated. This glycosylation likely impacts NS1 function as secretion of NS1 is dependent upon two N-glycosylation sites at Asn130 and Asn207, which are conserved amongst *Flaviviridae* family members. If either or both glycosylation sites present in the DENV or YFV NS1 protein were perturbed, viral titer and CNS invasion in intracranially infected mice decreased, suggesting that NS1 glycosylation may serve a pro-viral role in mediating viral infectivity and CNS entry [39,40].

### 3.4. Maintenance of Blood–Brain Barrier Integrity

The BBB is a highly selective, vascularized semipermeable barrier composed of brain microvascular endothelial cells, astrocytes, pericytes, and microglia joined by tight junctions. Its integrity prevents harmful or unnecessary molecules, viruses, and cells from reaching the brain, compromising its function. BBB integrity is maintained through interferons and cytokine signaling across the BBB interface, cellular receptors such as the TAM family of receptor tyrosine kinases, astrocyte regulation of endothelial cell growth factors, and matrix metalloproteins [2,41,42]. Viruses may enter the BBB through at least four previously described mechanisms: transcellular pathway, paracellular pathway, direct infection of the BBB endothelial cells, or the “Trojan horse” mechanism (reviewed in [43,44]). Various studies have examined the mechanisms by which RNA viruses may bypass the BBB and enter the CNS. For example, RABV enters the CNS through retrograde axonal transport into neurons, and NiV may enter the CNS through infection of leukocytes or direct infection of brain endothelial cells [45,46,47]. Although we know that viruses can enter the CNS and lead to neurological disease, for some of these viruses, the mechanisms of entry remain elusive. For example, Measles virus causes encephalitis and subacute sclerosing panencephalitis, and grey and white matter lesions have been observed in patients infected with MV [48]. Interestingly, how MV infects the CNS is not completely understood as MV-specific receptors have not been shown to be expressed within the CNS to date, warranting more research into these entry mechanisms [48].

Viruses, such as RABV, have evolved strategies to modulate BBB function. RABV is transmitted through the bite of infected animals. When individuals first present with symptoms and are treated with post-exposure prophylaxis, they are less likely to develop severe disease. However, once an individual develops neurological symptoms, the disease is thought to be fatal, although there are examples of patients who have survived infection or presumptive infection [49]. Rabies virus can replicate in the CNS without eliciting strong immune responses, suggesting it modulates immune responses rather well. In a study examining intradermal infection of several RABV strains in mice, including the attenuated CVS-F3 strain and the pathogenic SHBRV strain, Roy and colleagues found that attenuated RABV infection increased the BBB permeability of the cerebellum, allowing anti-RABV adaptive immune effectors to reach the CNS [50]. Recently, Long and colleagues found that BBB integrity was mediated through the function of the viral P phosphoprotein [51].

WNV also regulates BBB membrane integrity, although the literature is not as definitive on the contribution of IFNs to BBB integrity. Lazear and colleagues found that WNV infection of IFN-λ depleted mice results in increased WNV viral copies in the brain and spinal cord and increased BBB permeability [52]. IFN-λ signaling indirectly maintains the endothelial cell tight junctions in the brain and limits WNV infection [52]. The specific WNV proteins or tight junction proteins that may interact or be induced upon IFN-λ signaling would be an interesting topic for follow-up studies. The role of TLR3 during WNV infection has seemingly paradoxical roles [52]. WNV infection causes a physical breakdown of the BBB through regulation of host immune genes, including *TLR3* and *IFN-λ*. Six-to-ten week old TLR3 depleted mice are more resistant to lethal intraperitoneal WNV infection as there is less viral RNA and inflammation present in the brains of mice starting at 3 days post-infection, suggesting that WNV modulates TLR3 for pro-viral purposes [53]. However, another study by Daffis and colleagues found that TLR3 protects against WNV infection in the brain [54]. There are several differences between these two studies, such as route of infection, which help explain the conflicting results. These studies raise interesting questions, such as whether there are WNV proteins that antagonize TLR3 signaling in the CNS and whether the route of infection (intraperitoneal vs. intracranial) impacts immune responses or kinetics of immune responses. Further, TLR3 may in fact have dual roles during neurotropic virus infection and one role may have evolved against the other as neurotropic viruses continue to evolve. Regardless, future studies should delineate the viral proteins that may interact with TLR3 within the CNS using primary neuron cultures or neuron-like cell lines with various routes of infection and at multiples times post-infection.

### 3.5. Modulation of Autophagy

Autophagy is an evolutionarily conserved process used by cells to maintain cellular health and homeostasis [55]. Cells recycle unwanted organelles, cellular products, or foreign particles, such as viral proteins, through autophagic processes. Autophagy plays several essential roles during viral infection, including facilitating antigen processing and viral degradation [55]. Autophagy proteins can positively or negatively guide IFN production and inflammatory processes [56]. Regulation of autophagy is also observed in the context of the CNS, where autophagy is constitutively active to maintain neuronal health, as loss of autophagy can lead to neurodegeneration in mouse models [57,58]. During JEV infection in N2a mouse neuroblastoma cells, autophagy is induced and positively regulates JEV infection [59]. JEV-induced autophagy is negatively correlated with IFN-β production as silencing of autophagy-related genes led to the upregulation of cytokines, increased MAVS aggregation, and IRF3 activation [59].

Autophagy also serves a pro-viral role during infection with enterovirus A71 (EV-A71), a positive-sense, single-stranded RNA virus belonging to the *Picornaviridae* family. EV-A71 can cause hand, foot, and mouth disease, and in a subset of patients, can lead to CNS pathology. In human neuronal stem cells and IMR-32 neuroblastoma cells, EV-A71 infection induces the production of autophagosomes, acting as viral replication hubs [60]. Further, in brain tissues of EV-A71 infected mice, LC3-positive puncta increase, indicating autophagy induction in the brain.

### 3.6. Targeting Host Immune Proteins for Cleavage and Degradation

Viruses can target host immune proteins for cleavage and degradation using proteases encoded within their viral RNA genomes. For example, the flaviviruses ZIKV and WNV encode the protease NS2B-NS3, which cleaves host factors as well as the viral polyprotein. Although many of the studies examining host protease targets are performed in non-CNS cell types, these studies have identified several targets, including RIG-I, MAVS, TRIF, and STAT1 [61,62]. These proteins may also be likely viral targets within the CNS. One study, however, has explored host degradation by viral proteases within the CNS. Within neuronal SF268 cells, EV-A71 cleaved TRIF and MAVS. Interestingly, this cleavage does not result in reduced IFN-β induction [63]. In studies using non-neural cell lines, such as HeLa or rhabdosarcoma cells, cleavage of MAVS by the EV-A71 2A protease results in inhibition of IFN-β signaling [64]. The authors attribute these opposite findings to postponed expression of viral proteins in infected neural cells. A related enterovirus, EV-D68, also encodes the 2A and 3C proteases, which cleave several immune proteins, including TRIF and TRAF, inhibiting IFN-β induction [65,66]. To our knowledge, there are no published studies that have examined these EV-D68 cleavage events within CNS cell lines. Thus, it would be interesting to see if the differences observed in IFN-β induction in the EV-A71 studies are also seen with EV-D68.

### 3.7. Regulation of microRNAs and Host or Viral Gene Expression

MicroRNAs (miR), short single-stranded RNAs, regulate the expression of host genes by binding to the 3′UTR of their target genes. miRs function in neuronal development, neuronal migration, and in CNS inflammation regulation [67]. As such, viruses can antagonize miR expression or function for neurological disease. miR antagonism by viruses is perhaps most studied using JEV; however, this is still relatively underexplored. Several miRs and their host targets have been identified in various mouse models or neuronal cell lines and are outlined in Table 2. During JEV infection of human microglial cells, miR-146a targets TRAF6, IRAK1, IRAK2, and STAT1 to inhibit IFN signaling [68]. Similarly in mouse cortical neurons and granule cell neurons, JEV infection induces the expression of miR-132, limiting STAT1 activation [69]. IRF1-mediated signaling is also blocked by JEV infection-induced expression of miR-15b [70].

Viruses can directly alter the expression of host genes. During VEEV infection of neuro-2a cells, the viral sP protein induced the shutoff of host macromolecular synthesis [74]. This resulted in suppression of IFN-α/β and ISG activation [74]. Several other RNA viruses, such as those belonging to the SARS family, can inhibit the expression of host genes. For example, the SARS-CoV viral protein Nsp1 blocks the transcription of IFN-β and related genes [75]. Future work should explore if other viruses, such as other encephalitic viruses, alter host gene expression within the CNS.

### 3.8. Escape of Adaptive Immune Responses and Molecules

Adaptive immune responses may function to limit viral titers and associated pathology. When immunologically naïve mice are infected with sera from mice previously infected with EV-D68, these naïve mice were protected from developing paralysis, underscoring the important role of antibody responses in reducing neurological disease (reviewed in [76]). As such, viruses may also antagonize adaptive immune responses during infection, acting as an additional defense strategy to aid in persistence within their hosts. RABV upregulates the expression of HLA-G1 and HLA-G5/G6 in neurons [77]. This is thought to prevent T cell migration because of an increase in specific apoptosis of T cells, upregulating the expression of Fas-L and B7-H1 and triggering the exhaustion of CD3/8+ T cells [77]. Collectively, these events are considered immunosubversive. Relatively little else is known about viral antagonism of adaptive immune responses specifically within the CNS. However, given that at least one virus utilizes such a strategy, there is a likelihood that other neurotropic viruses do as well, highlighting the necessity of studying T cell responses and other adaptive immune processes within the CNS during neurotropic virus infection.

### 3.9. Amino Acid Mutations Driven by Adaptation

Most RNA viruses contain an error-prone RNA polymerase and lack proofreading mechanisms, leading to high mutation rates [78,79]. This in turn drives RNA virus population diversity and adaptability in new environments, leading to survival of the most evolutionarily fit viruses [80]. RNA viruses that mutate to adapt to external pressures, such as new hosts or new host antiviral immune strategies, will outcompete those that do not. This creates the potential for emerging viruses or viruses with an evolutionarily adapted capability to enter the CNS. There is limited research into viral adaptive mutations within the CNS. However, given that in cell culture studies or in sequencing of infected patient samples, several RNA viruses can undergo adaptive mutations [81,82,83], mutations arising that are beneficial for either entering the CNS or evading responses within the CNS are likely. One study has found that in A129 mice, an A188V mutation within the Zika virus NS1 protein increased ZIKV loads in brain tissue [84]. This mutation promoted the binding of NS1 to TBK1, limiting IFN-β induction [84]. This study highlights the continued need for collecting and sequencing patient samples to identify amino acid polymorphisms that may be beneficial for increased immune evasion or CNS neuroinvasion.

## 4. Conclusions and Future Perspectives

In this review, we have highlighted the ways in which neurotropic RNA viruses modulate immune responses in the CNS for pathogenesis, replication, and infection. Strategies include regulation of interferon induction and responses, sequestration of host immune proteins, modulation of post-translational modifications and related machinery, adaptive amino acid mutations, regulation of autophagy, maintenance of blood–brain barrier integrity, targeting of host proteins for degradation, and controlling adaptive immune responses. There are likely several additional mechanisms that viruses may use to antagonize responses that have been not explored. Research into emerging viruses that cause neurological disease is a burgeoning field, with new roles for viruses causing neurological diseases being identified. Indeed, two recent pioneering studies linked Epstein–Barr virus as the cause of multiple sclerosis [85,86], further emphasizing the importance of research into viral neurotropism and immune regulation.

Tools for reverse genetic studies and recombinant virus generation have greatly aided both in vitro and in vivo insight into viral pathogenesis and immune evasion strategies. Yet, relatively few studies have investigated immune evasion strategies in cell types of the CNS, such as the relatively under-characterized oligodendrocytes, in neuron-like cell lines, such as the neuroblastoma cell lines SH-SY5Y or N2a, or in astrocyte cell lines such as A735. A previous study found that neurons from specific regions of the brain initiate differential immune response programs to RNA virus infections [14]. This finding emphasizes the importance of comparative analysis of virus infection or virus protein over-expression in several CNS cell types or cell lines. Although it is becoming more evident that RNA viruses antagonize immune responses within the CNS, many of the mechanisms of suppression and evasion are poorly characterized. In vivo analysis of ISG regulation, antagonism, and induction in the CNS should also be explored.

The role of microglia during neurotropic virus infection is seemingly contradictory, particularly during WNV infection. Microglia can serve a protective role during WNV disease in animal models or may also help facilitate WNV entry and replication and induce neurological pathogenesis. It is also likely that microglia may serve these dual roles during infection with other neurotropic flaviviruses.

It is likely that new viruses will emerge with the capacity to infect CNS tissue or that previously circulating viruses may evolve to become neurotropic. Such an example is observed with SARS-CoV-2, the causative agent of one of the deadliest pandemics. There is increasing evidence that SARS-CoV-2 may be neurotropic, as neurological symptoms have been reported in patients [87]. Additionally, RNA-sequencing analysis and transgenic mouse models have revealed that the SARS-CoV-2 receptor, ACE2, is present in CNS cells [88,89], further supporting the neurotropic potential of SARS-CoV-2. Mechanistic exploration of SARS-CoV-2 neuroinvasion and evasion of CNS immune responses will be an important area of future research.

The majority of research on viral immune evasion strategies has been performed in non-CNS cell lines, such as HeLa cells or HEK-293T cells, and there are several reasons for this. First, these cells lines are more accessible than primary neurons or isolated CNS cells from mouse models. Second, many of these emerging neurotropic viruses are biosafety level 3 or 4, limiting research into their evasion strategies during full-length infection. Last, establishment of animal models and protocols for isolating pure CNS cell types from mouse nervous tissue can be challenging. Yet, many important discoveries and host-viral immune interactions have been identified using non-CNS cells during virus infections. These factors underscore the importance of research characterizing immune modulation strategies by neurotropic viruses using non-CNS cells and in vitro assays. Perhaps the immune evasion strategies utilized in non-CNS cells may be how some neurotropic viruses evade signaling in the initial infected cells to enter the CNS. Once infection is established in the CNS, viruses may utilize these same strategies or deploy different ones to adapt to the new cellular environment. However, looking towards the future and if resources allow, we can begin to address these difficult questions in the context of animal models and in CNS tissue, harnessing the power of both in vitro techniques and in vivo applications.

## Figures and Tables

**Table 1:** Central nervous system (CNS) manifestations during neurotropic virus infection.

Virus	CNS Manifestation
PV	Polio (paralysis)
EV-D68	Acute flaccid myelitis, encephalitis
EV-A71	Meningitis, acute flaccid paralysis, hand-foot-and-mouth disease (non-CNS)
Coxsackievirus A16	Encephalitis
RABV	Rabies (anxiety, hydrophobia, coma), encephalitis
ZIKV	Microcephaly, meningoencephalitis, Guillain-Barré syndrome, non-specific acute febrile illness
DENV	Fever, encephalitis, meningitis
MuV	Sensory motor axonopathy, meningitis
MV	Encephalitis, brain edema, myelitis, sclerosing panencephalitis (SSPE), measles (non-CNS)
NiV	Encephalitis, meningitis
JEV, VEEV, WEEV, EEEV	Encephalitis, meningitis
LACV	Encephalitis, meningitis, non-specific febrile illness
SARS-CoV-2	Encephalitis, acute necrotizing encephalopathy, meningitis, acute cerebrovascular disease, confusion

**Table 2:** Viral protein host targets for immune antagonism in the CNS.

Virus.	Viral Protein(s)	Host Target	Cell Type(s)	Reference
WNV	NS1, NS2A	IFN-β and NF-ⲕB signaling	BE(2)-C/m (neuroblast cell line)	[9]
ZIKV	NS3	14-3-3ε and 14-3-3η signaling	SVGA (immortalized human astrocyte cell line)	[25]
RABV	Capsid	TLR3	NT2-n, SK-n-SH, Ntera-2clD/1	[22]
JEV	Unknown	miR-15b targeting of RNF125	U251 (human astrocytoma cell line), mouse brain, BV-2 (mouse microglia cell line)	[70]
	Unknown	miR-301a targeting of IRF1 responses	HT22 (immortalized mouse hippocampal neuronal cell line)	[71]
	NS5	Suppressor of cytokine signaling (SOCS3)	Mouse brain	[72]
	Unknown	miR-146a targeting of TRAF6, IRAK1, IRAK2, and STAT1	Human microglial cells	[68]
	Unknown	miR-132 targeting of p300 co-activator of STAT1	Mouse cortical neurons and mouse granule cell neurons	[69]
	Unknown	miR-432 targeting of SOCS5	CHME3 (human microglial cells)	[73]
VVEEV	sP	Macromolecular shutoff	Neuro-2a	[74]
Enterovirus A71	3C	TRIF, MAVS	SF268 (human glioblastoma)	[63]
Nipah virus	P protein	STAT1, IFN-β signaling	Ferret model brain	[10]

## Data Availability

Not applicable.
